# Supplementary material for: Genetic Deletion of Thorase Causes Purkinje Cell Loss and Impaired Motor Coordination Behavior
Source: Cells. 2023 Aug 10;12(16):2032. doi: 10.3390/cells12162032 (PMC10453921; doi:10.3390/cells12162032)
Supplement: Supplementary file 1 [file cells-12-02032-s001.zip › cells-2426724-supplementary.pdf]

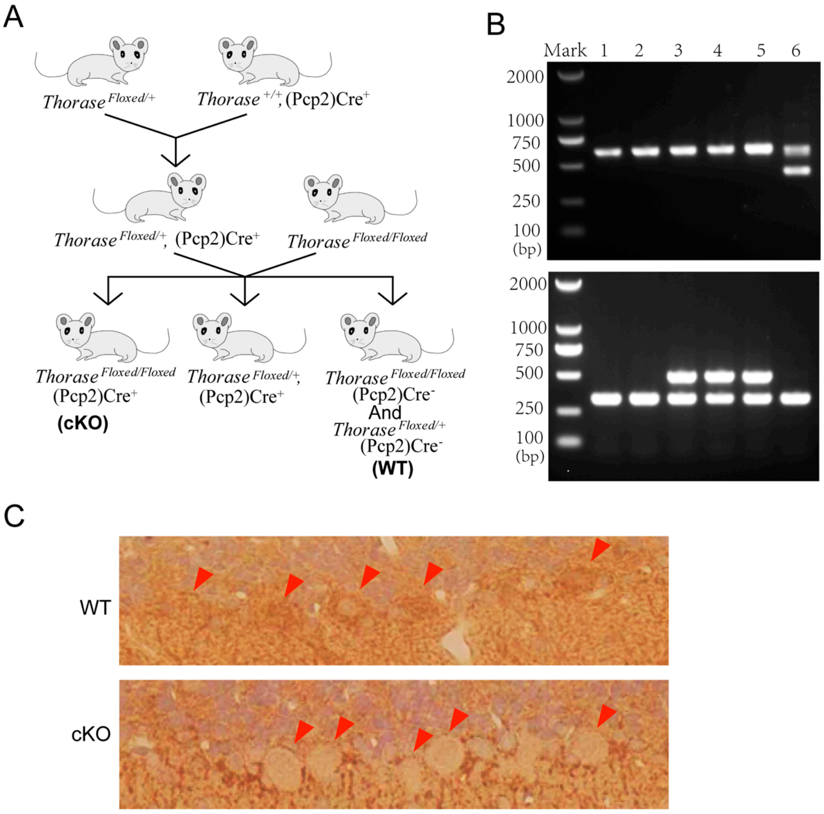

Figure S1. Preparation of Thorase conditional knockout mice lacking Thorase in Purkinje cells. Mouse mating strategy and identification of Thorase cKO mice. (**A**) Schematic diagram of Thorase cKO mouse breeding. (**B**) Typical images of Thorase cKO mouse genotyping by PCR. (**C**) Immunohistochemistry to detect Thorase expression in the Purkinje cells in WT and cKO mice with antibodies against Thorase.
